# The Neuroanatomy of the Reticular Nucleus Locus Coeruleus in Alzheimer’s Disease

**DOI:** 10.3389/fnana.2017.00080

**Published:** 2017-09-19

**Authors:** Filippo S. Giorgi, Larisa Ryskalin, Riccardo Ruffoli, Francesca Biagioni, Fiona Limanaqi, Michela Ferrucci, Carla L. Busceti, Ubaldo Bonuccelli, Francesco Fornai

**Affiliations:** ^1^Section of Neurology, Pisa University Hospital, Department of Clinical and Experimental Medicine, University of Pisa Pisa, Italy; ^2^Department of Translational Research and New Technologies in Medicine and Surgery, University of Pisa Pisa, Italy; ^3^I.R.C.C.S. I.N.M. Neuromed Pozzilli, Italy

**Keywords:** neurofibrillary tangles, basal forebrain nuclei, phospho-Tau, amyloid, mild cognitive impairment, pre-clinical AD

## Abstract

Alzheimer’s Disease (AD) features the accumulation of β-amyloid and Tau aggregates, which deposit as extracellular plaques and intracellular neurofibrillary tangles (NFTs), respectively. Neuronal Tau aggregates may appear early in life, in the absence of clinical symptoms. This occurs in the brainstem reticular formation and mostly within Locus Coeruleus (LC), which is consistently affected during AD. LC is the main source of forebrain norepinephrine (NE) and it modulates a variety of functions including sleep-waking cycle, alertness, synaptic plasticity, and memory. The iso-dendritic nature of LC neurons allows their axons to spread NE throughout the whole forebrain. Likewise, a prion-like hypothesis suggests that Tau aggregates may travel along LC axons to reach out cortical neurons. Despite this timing is compatible with cross-sectional studies, there is no actual evidence for a causal relationship between these events. In the present mini-review, we dedicate special emphasis to those various mechanisms that may link degeneration of LC neurons to the onset of AD pathology. This includes the hypothesis that a damage to LC neurons contributes to the onset of dementia due to a loss of neuroprotective effects or, even the chance that, LC degenerates independently from cortical pathology. At the same time, since LC neurons are lost in a variety of neuropsychiatric disorders we considered which molecular mechanism may render these brainstem neurons so vulnerable.

## Introduction

Alzheimer’s Disease (AD) affects more than 45 million of people worldwide (Alzheimer’s Disease International, 2015). Despite research efforts, key molecular mechanisms of disease remain uncertain. Milestones in AD pathology consist in alterations of the cytoskeleton-associated Tau protein along with abnormal β-amyloid (Aβ) depositions. A long-lasting time interval exists since the onset of early pathological alterations until the appearance of cognitive deterioration. In fact, a sub-clinical phase, in which cortical AD pathology occurs in the absence of a frank cognitive impairment is documented. Such a stage, in which a cognitive intact subject already bears AD markers is defined “pre-clinical asymptomatic at-risk for AD” (Dubois et al., 2014). We wish to emphasize that, despite being a fascinating hypothesis, the causal relationship between Tau pre-clinical pathology and later cognitive deterioration remains to be established. Cortical pathology consists of: (i) argyrophilic structures formed by abnormal intracellular aggregates of phospho-Tau (P-Tau) fibrils, known as neurofibrillary tangles (NFT); and (ii) extracellular aggregates of Aβ known as amyloid plaques (Hyman et al., 2012; Montine et al., 2012).

An updated hypothesis considers six stages of NFT pathology. At stage I, a low amount of Gallyas-positive NFT is present, mainly within the trans-entorhinal region, which corresponds to the peripheral entorhinal cortex (Braak and Braak, 1991 or the parahippocampal gyrus facing the fusiform gyrus with the collateral sulcus interposed (Taylor and Probst, 2008). At stage I scattered NFT can be detected also within entorhinal mesocortex, CA1, dorsomedial thalamus and basal forebrain nuclei (Braak and Braak, 1991). At stage II the trans-entorhinal cortex is more affected, while the entorhinal mesocortex is consistently affected along with CA1 and prosubiculum; dorsomedial thalamus and basal forebrain nuclei appear as at stage I. At stage III, NFT densely cluster in the superficial layer of both trans-entorhinal and entorhinal cortex, while hippocampal involvement extends towards subiculum. Isocortex (neocortex) is spared apart from a few NFT within associative ventral areas. At stage IV all layers of trans-entorhinal and entorhinal cortex are filled with NFT, while the CA1 region features ghost tangles, and the basal frontal and insular isocortex are involved. At this stage also the amygdala and endopiriform nucleus feature NFT. At stage V, the parasubiculum and the whole hippocampal formation are involved massively in a continuum with trans-entorhinal and entorhinal cortex. Remarkably, CA2 is mostly spared along with dentate gyrus (Dudek et al., 2016). This is reminiscent of hippocampal damage following seizures and ischemia (Giorgi et al., 2008). At this stage the associative isocortex is widely affected. Finally, at stage VI, primary sensory and motor areas are affected. In most patients stage I and II are rather asymptomatic, while during stage III memory impairment may appear.

Aβ aggregates follow an opposite spreading direction (Thal et al., 2002) since they first appear in the isocortex (phase 1) and later on within allocortex (phase 2), then downstream to diencephalon (phase 3) and furtherly towards brainstem nuclei including substantia nigra (phase 4). In the last step Aβ extends to caudal reticular nuclei including locus coeruleus (LC; phase 5).

The spreading of NFT through interconnected brain regions may occur trans-synaptically via a prion-like transmission of Tau (Mohamed et al., 2013; Goedert, 2015). These synaptic mechanisms of degeneration are supposed to start caudally within the iso-dendritic core of the reticular formation. In fact, LC and other reticular nuclei feature an impressive collateralization allowing a single axon to innervate multiple brain regions making these cells ideal spreading vectors. A variety of brainstem reticular nuclei features NFT. Similarly, when considering cortical neurons long-projecting pyramidal cells of layer V or hippocampal pyramidal cells appears more vulnerable to degeneration (Ovsepian et al., 2016; Schaeffer et al., 2017). The present review emphasizes the role of LC as a powerful brainstem nucleus, which projects mono-synaptically to all cortical regions (Nagai et al., 1981). Nonetheless, while writing this article we already feel the limits of such a LC-centered hypothesis, since other nuclei such as the rostral dorsal raphe, the parabrachial nuclei and the pedunculopontine nucleus are important as well. Thus, the LC should be regarded more as a paradigm, rather than the unique anatomical entity, which connects NFT pathology from the brainstem to the cortex.

The trans-synaptic spreading of Tau from LC to other brain regions may occur “a rebour” (i.e., in opposite directions) by hippocampal injections of Tau fibrils, which produce a frank pathology down to the LC (Iba et al., 2015).

## Tau Pathology Occurs Early within Brainstem, Cortical-Projecting Nuclei

Recent studies indicate early impairment of LC with potential outcomes on preclinical staging and neurobiology of disease (Braak et al., 2011). In detail, stereology consistently evidenced that the number of LC neurons negatively correlates with AD pathology. Pioneer studies by Tomlinson et al. (1981), Bondareff et al. (1982) and Mann et al. (1982, 1984) described a reduction of LC neurons in AD patients. A few years later Chan-Palay and Asan (1989) proposed a rostro-caudal gradient of neuronal loss within LC of AD patients, differently from PD patients where LC is uniformly affected. In a large series of non-selected brains (*N* > 2300), Braak et al. (2011) by using P-Tau antibodies detected “pre-tangle material” (negative for Gallyas reaction) within LC of almost all the brains in the absence of Tau-related pathology in the trans-entorhinal region. This emphasizes the occurrence of subcortical deposition of Tau in the absence of NFT in any cortical area (less than stage I). Depending on the severity of brainstem involvement, a progressive staging (“a”, “b”, “c”) up to early cortical recruitment (stages “1a” and “1b”) is described (Table 1). These stages are age-related, being stages “a-c” present solely at age 20–30, while stages > I–II being detected only after the age of 40. These findings lead to the following statements: (i) LC features abnormal Tau deposits before a frank neuronal loss; (ii) Tau deposits in LC occur decades before the average onset of cortical AD pathology; (iii) P-Tau accumulation is likely to be key in the process of NFT formation; and (iv) Tau alterations anticipate dementia. The latter observation challenges quite directly the classic amyloid cascade hypothesis, according to which, an impairment in Aβ-pathway may trigger Tau pathology (Hardy and Selkoe, 2002; Jack et al., 2010). In contrast, one might hypothesize that early Tau pathology predisposes to Aβ accumulation, which in turn exacerbates Tau pathology. Very recently, Theofilas et al. (2017) applied up-to-date stereology to brains at various disease stages and showed a two-fold increase in P-Tau positive LC neurons from pre-stage 1 to stage I, with a positive correlation between the number of P-Tau positive cells in the LC and disease stage. They also found that volume in the rostral part of LC decreases already from pre-stage 1 to stage I. The number of LC neurons decreases form stage II onward. In another study in asymptomatic patients, Andrés-Benito et al. (2017) confirmed these data. On the other hand, these studies are grounded on static descriptions, where a dynamic mechanism remains at hypothetical level. The cross-sectional evidence reported in these studies does not represent a proof of principle for an actual spreading of pathology. Thus, casualty cannot be ruled out and a supreme vulnerability of LC neurons represents a sort of background noise occurring in a number of neurological disorders encompassing AD, PD, seizures, multiple system atrophy, Down syndrome and many others (Fornai, 2007; Phillips et al., 2016). In fact, the loss of brain norepinephrine (NE) levels characterizes a number of neurological patients and involves multiple brain areas (Gesi et al., 2000; Giorgi et al., 2003, 2004; Marien et al., 2004; Fornai et al., 2011; Ruffoli et al., 2011; Pifl et al., 2012, 2013) and various experimental neurological disorders (Fornai et al., 1995a,b, 1997a,b, 1998, 1999, 2007; Siciliano et al., 1999; Soldani and Fornai, 1999; Ferrucci et al., 2002, 2013; Fulceri et al., 2004; Giorgi et al., 2006; Weinshenker et al., 2008).

**Table 1:** Staging of neurofibrillary tangles (NFT-related) pathology in Locus coeruleus (LC).

	Subcortical pretangles stages	a	P-Tau accumulation within the axon hillock of brainstem reticular neurons, mostly LC neurons.
		b	P-Tau accumulation extended further into LC cell bodies.
Pretangle Stages		c	Involvement of other reticular (or reticular-related) ascending nuclei (e.g., dorsal raphe nucleus, nuclei of the basal forebrain).
	Cortical pretangles stages	1a	Pre-tangle material in LC axons within the trans-entorhinal and entorhinal regions.
		1b	Pre-tangle inclusions within pyramidal cells of the trans-entorhinal region connected with NFT positive axons.

## The Neuroanatomy of LC in AD

LC is a tube-like shaped group of NE neurons placed in the upper part of the pons; it is composed of medium-sized neurons (Brodal, 1981). LC extends rostrocaudally for approximately 16 mm (German et al., 1988; Baker et al., 1989; Halliday, 2004). These neurons send their branched axons to innervate the entire cerebral cortex (Nagai et al., 1981). Thus, by releasing NE through “bouton en passage” (or synaptic varicosities), LC modulates the activity of several cortical areas. In fact, LC modulates sleep/wake cycle, learning and memory, early gene expression and neuroprotection (Fornai et al., 1990; Foote et al., 1991; Cirelli et al., 1996; Cirelli and Tononi, 2000; Giorgi et al., 2003, 2004; Aston-Jones, 2005; Aston-Jones et al., 2007; Fornai, 2007; Giorgi et al., 2008; Weinshenker et al., 2008; Sara, 2009; Fornai et al., 2011; Counts and Mufson, 2012; Ferrucci et al., 2013). LC mostly innervates limbic cortex compared with neocortex (here defined as isocortex, Jones and Yang, 1985; Loughlin et al., 1986; Giorgi et al., 2003). The ventral part of caudal LC sends projections even to lower medulla and spinal cord, and regulates autonomic functions (Ward and Gunn, 1976), while neurons which innervate the allocortex are placed in the rostral part of the nucleus (Ward and Gunn, 1976; Loughlin et al., 1986).

The projection of LC neurons to the cortex may occur monosynaptically (Fallon et al., 1978; Nagai et al., 1981; Harley, 1987), via the allothalamus (Krout et al., 2002; Garcia-Rill et al., 2013), or through basal cholinergic nuclei. These are severely involved in AD (Davies and Maloney, 1976; Coyle et al., 1983; Sassin et al., 2000). In particular, degeneration of Ch1 and Ch2 neurons is responsible for the loss of septo-hippocampal connections leading to memory impairment (Gertz et al., 1987; Brayda-Bruno et al., 2013). The Ch4 (nucleus basalis of Meynert) nuclear complex, which is divided into various subfields (Mesulam, 2013) and receives dense NE projections from LC degenerates as well (Mesulam et al., 1983a,b, 1984; Mesulam and Geula, 1988; Smiley and Mesulam, 1999; Haghdoost-Yazdi et al., 2009). The Ch4 sends a strong cholinergic input to allocortical and mesocortical areas (Mesulam et al., 1986). In line with this, the joint involvement of cholinergic and NE nuclei encoding motivational salience in spatial attention explains the loss of motivational information and spatial attention that is critically lost in dementia (Mohanty et al., 2008; Sara, 2015; Chandler, 2016). Another limbic region innervated by LC is the amygdala (Asan, 1998) which is involved in AD (Knafo, 2012). The entorhinal and trans-entorhinal cortex as well as the hippocampal formation are monosynaptically innervated by LC (Fallon et al., 1978; Room and Groenewegen, 1986; Harley, 1987). Entorhinal cortex is involved in memory consolidation and retrieval (Dolcos et al., 2005; Bott et al., 2016; Cho et al., 2016; Fayed et al., 2017). Within hippocampus, LC axons innervate the stratum radiatum of CA1 and stratum lucidum of CA3 (Melander et al., 1986; Moudy et al., 1993). Despite a preferential limbic innervation which is in line with AD pathology, a fine anatomical analysis of the cytotectonic pattern of LC innervation within allocortex does not provide any site-specificity which may justify a preferential damage to layer V long-projecting neurons which occurs in AD. In fact, the pattern of catecholamine innervation is rather uniform in the various layers of the allocortex (apart from the scarce density in layer I; Gaspar et al., 1989). This rules out a site-specific, disease-related synaptic contact with those cortical cells (pyramidal) which are mostly affected in AD. Thus, there is no anatomical overlapping between the fine pattern of NE innervation and the fine pattern of neuropathology within various cortical layers. Such a discrepancy also applies to NE innervation of isocortical (neocortical regions; Gaspar et al., 1989). Remarkably, albeit primary sensorimotor regions are affected only at latest AD stages, they possess the highest NE innervation among isocortical regions (Gaspar et al., 1989). Thus, we have to rule out a point-by-point matching between NE axons and degenerating cortical neurons, which in turn rules out a trans-synaptic disease spreading between NE fibers and cortical neurons. This calls for considering alternative mechanisms, which may link a damage to LC axons to cortical degeneration. In fact, when analyzing the pattern of NE release rather than a point-by-point connection between pre- and post-synaptic sites a massive extra-synaptic neurotransmitter diffusion takes place. In fact, NE release allows the amine to affect close glial cells and brain vessels (Stone and Ariano, 1989; Toussay et al., 2013).

## The Mechanisms Which May Link LC Degeneration to AD

In early studies LC neurons were quantified without stereology. This explains a great variability in LC cell counts even within control subjects.

Modern stereology confirms LC neuronal loss (Grudzien et al., 2007; Counts and Mufson, 2010; Kelly et al., 2017). P-Tau positive LC neurons are associated with a dramatic reduction of synaptophysin-positive perineural dots, which leads to a loss of synaptic connectivity, a sort of anatomical de-afferentiation of these neurons from activating inputs (Andrés-Benito et al., 2017). These authors found over-expression of α2-adrenergic receptors at stage I, while these receptors were reduced at stage IV in the hippocampus (Andrés-Benito et al., 2017).

Altogether these pieces of information suggest that P-Tau accumulates within LC very early, producing subtle, yet potentially relevant, functional alterations affecting NE release in target areas.

P-Tau pre-tangles accumulate within pyramidal cortical limbic cells after NE axon terminals are filled with pre-tangles themselves (pre-tangle stage 1b). Later on, the occurrence of NFT inclusions in trans-enthorinal cortical neurons parallels the increase of P-Tau inclusions within LC axons, and spreads to other cortical structures (Braak et al., 2011). At stage III, when prodromal form of AD may appear, P-Tau burdens in LC neurons are severe enough to cause neuronal death. Early pre-tangle impairment of LC leads to an altered pattern of NE release within target regions. In baseline conditions NE release is finely tuned, and it is regulated by strong afferents from different sensory inputs (Samuels and Szabadi, 2008) which are altered early in the course of disease (Andrés-Benito et al., 2017), making surviving LC cells less effective. The specific anatomy of LC neurons makes them ideal vectors throughout the CNS. In fact, Tau aggregates may pass trans-synaptically from LC axons to cortical neurons and from one cortical region to another and back again (Mohamed et al., 2013; Goedert, 2015).

At advanced stages, the loss of NE neurons enhances the accumulation of Aβ in cortical regions: this in line with experimental evidence. In fact, the neurotoxin DSP-4 (which causes selective degeneration of LC axons, Fornai et al., 1996) enhances Aβ plaque accumulation in the brain (Heneka et al., 2006). Thus, the loss of NE appears to directly foster Aβ plaque accumulation (Kalinin et al., 2007) and LC degeneration alters microglial activity, which may indirectly induce amyloid mismetabolism (Heneka et al., 2010). These changes correlate with cognitive impairment (Jardanhazi-Kurutz et al., 2011).

Again, a damage to LC produces a lack of neuroprotection. In fact β2-receptor stimulation is key in activating autophagy (Aránguiz-Urroz et al., 2011; Farah et al., 2014; Wauson et al., 2014) which removes P-Tau from the trans-Golgi network.

A recent study demonstrates an accumulation of P-Tau pyramidal cell bodies from AD patients correlates with alterations of the Golgi apparatus (Antón-Fernández et al., 2017).

**Figure 1:**
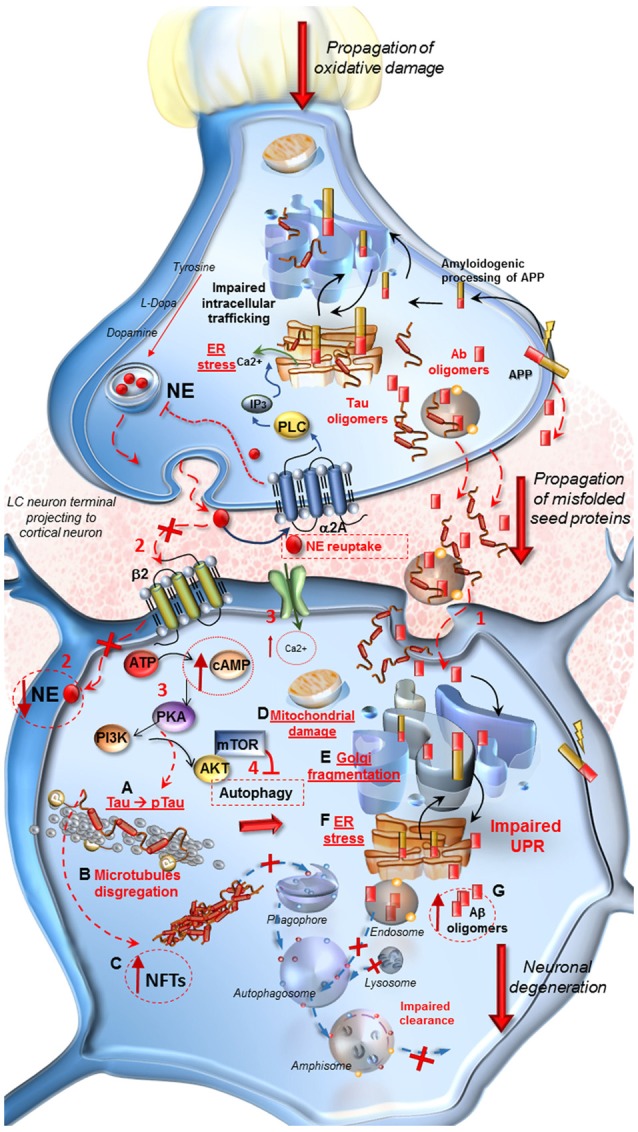
Molecular events occurring in Alzheimer’s Disease (AD) following locus coeruleus (LC) norepinephrine (NE) loss and autophagy impairment. *At LC pre-synaptic terminal*. Decreased expression of β2-adrenergic receptors in the pre-synaptic terminal leads to autophagy suppression. Oxidative stress, NE decrease and IP3 all contribute to endoplasmic reticulum stress and impairment of proteins processing, folding and trafficking in the trans-Golgi network. Aβ oligomers seeds, deriving from an amyloidogenic cleavage of APP, as well as Tau protein, are engulfed into vesicles and released into the axo-somatic synaptic cleft. *At post-synaptic cortical neuron*. **(1)** Aβ and Tau seeds are spread into the synaptic cleft and internalized into the cell body of the cortical neuron. **(2)** The loss of β2-adrenergic receptors (β2R) stimulation decreases autophagy. Thus the lack of NE, cannot exert its protective effect in the cell, it drives a cascade of detrimental intracellular effects instead. **(3,4)** Ca^2+^ entry into the cell, increase of cAMP levels, activation of PKA and PI3K/AKT/mTOR pathway lead to a further inhibition of autophagy. **(A–G)** PKA hyper-phosphorylates Tau protein **(A)** which leads to microtubules disgregation **(B)** and to the formation of neurofibrillary tangles (NFTs). **(C)** These effects translate into mitochondrial damage **(D)**, Golgi fragmentation due to the inactivation of associated binding proteins **(E)** and ER stress **(F)**. These events disrupt the Unfolded Protein Response (UPR) which does not provide for a proper intracellular trafficking, processing and sorting of misfolded Aβ, leading to further increase of harmful oligomers into the cell **(G)**. Misfolded proteins are internalized into the endosomal compartment but autophagy impairment does not allow their removal and fosters trans-synaptic propagations.

A lack of β2-mediated autophagy stimulation both on pre-synaptic LC axons and post-synaptic cortical neurons might be key in triggering NFT (Figure 1). Similarly, the loss of β-receptors stimulation leads to a decrease in growth factors expression (Follesa and Mocchetti, 1993) and permanently alters immediate-early-genes expression (Cirelli and Tononi, 2000). Most directly, NE activation of neurotrophic pathways was shown to protect against neuronal amyloid toxicity (Counts and Mufson, 2010). Again, NE innervations, which occur following a volume transmission, may alter a variety of anatomical structures including astrocytes, glial cells and blood vessels which can be reached through a paracrine diffusion of NE (Stone and Ariano, 1989; Stone and John, 1991; Gesi et al., 2000; Marien et al., 2004). In this way, the neurovascular unit can be affected at multiple levels by a lack of NE innervation (Lecrux and Hamel, 2016). For instance, LC activation increases brain perfusion which triggers over-activity of multiple cortical cells (Toussay et al., 2013). This may contribute to explain why in vascular dementia Tau and amyloid accumulation occur as well (Mendel et al., 2010; Day et al., 2015; Saito et al., 2015). On the other hand, one should consider the chance that co-transmitters released in concomitancy with NE may participate to neuroprotective effects. This is the case of adenosine, which is known to be neuroprotective, as well as galanine, and others (Crawley, 1993, 1996; Stone et al., 2009; Huang et al., 2011). Just like NE these co-transmitters act by increasing autophagy, which removes both Tau and Aβ, while inducing a higher perfusion rate in the neurovascular unit and by modulating Ach release.

The time window, lasting years, from the onset of P-Tau accumulation in the LC to the onset of LC neuronal loss, might be the best timing for trying to halt AD onset and progression (Mather and Harley, 2016; Ehrenberg et al., 2017). Thus, molecular mechanisms and sub-cellular sites, which first accumulate P-Tau under the effects of NE loss, need to be investigated. The high vulnerability of the isodendritic cells of the LC reticular neurons relies on the archaic nature of these cells which are preserved to transmit multimodal activities and do not develop effective protective mechanisms (Theofilas et al., 2015; Gambardella et al., 2017). In fact, these neurons are sensitive to a wide spectrum of stressful stimuli. This implies the convergency of a number of excitatory pathways making these cells a paradigm for critical overstimulation within the brain. A balanced stimulation of the autophagy machinery, which removes both Tau and Aβ could be an important step in relenting the neurobiology of disease.

## Author Contributions

FSG wrote the article. LR contributed to the writing of the article and made artwork. RR provided a critical review of the literature. FB contributed to the artwork. FL contributed to the writing of the article and conceptualization. MF contributed the writing of the article. CLB contributed to the review of the literature. UB provided a review of the literature. FF wrote the article and provided a critical review of the whole manuscript.

## Conflict of Interest Statement

The authors declare that the research was conducted in the absence of any commercial or financial relationships that could be construed as a potential conflict of interest.
